# The efficacy of duration of prophylactic antibiotics in transrectal ultrasound guided prostate biopsy

**DOI:** 10.1590/S1677-5538.IBJU.2014.0419

**Published:** 2015

**Authors:** Volkan Bulut, Ali Feyzullah Şahin, Yavuz Balaban, Muammer Altok, Rauf Taner Divrik, Ferruh Zorlu

**Affiliations:** 1 Department of Urology, Akyazi Public Hospital, Sakarya, Turkey; 2 Department of Urology, Şifa University Medicine School, İzmir, Turkey; 3 Department of Urology, Kahta Public Hospital, Adiyaman, Turkey; 4 Department of Urology, Suleyman Demirel University Medicine School, Isparta, Turkey; 5 Department of Urology, Tepecik Training Hospital, İzmir, Turkey

**Keywords:** Antibiotic Prophylaxis, Prostate, Biopsy, Ultrasound, High-Intensity Focused, Transrectal

## Abstract

**Introduction:**

We aimed to evaluate the efficacy of the duration of prophylactic antibiotic administration in patients undergoing transrectal ultrasound (TRUS) guided biopsy.

**Material and Methods:**

A total of 367 patients undergoing a prostate biopsy between September 2007 and June 2009 was reviewed retrospectively and divided into 2 groups according to prophilaxy: oral ciprofloxacin (750 mg every 12 hours) for 3 or more days in Group-1 and single day in Group-2. Demographic characteristics of patients, symptoms, PSA values, IPSS scores, prostate sizes, pathologic results and complications were compared between the groups.

**Results:**

The mean age of all patients was 63.92 years and the mean PSA was 13.61ng/ dL. The pre-biopsy mean IPSS score was 12.47 and mean prostate volume 52.53 mL. For 78.2% of patients the current biopsy was their first biopsy. Cancer detection rate was 24.2%. Fever was observed in 3 (1.2%) patients in Group-1 and 5 (4.0%) patients in Group-2. Local infections occurred in 2 (0.8%) and 1 (0.8%) patients respectively in Groups 1 and 2. Acute prostatitis was observed in only 1 (0.8%) patient in Group-2. Accepted after revision: None of the patients developed septicemia or other serious infection. There was no statistically significant difference in terms of fever, local infections (epididimitis, orchitis, etc.) and acute prostatitis.

**Conclusions:**

In a selected patient population single dose prophylaxis with ciprofloxacin can be safely administered compared to other regimens of 3 or more days. Increasing the duration of antibiotic prophylaxis does not decrease infectious complications.

## INTRODUCTION

The necessity of antibiotic prophylaxis before biopsy is well established by randomized, controlled trials in literature. Despite the existing consensus, the type, dosage and duration of treatment have not become clear (1). It is known that antibiotic prophylaxis before transrectal prostate biopsy reduces infectious complications (2). Up to date both oral and intravenous administrations of several different prophylactic regimens have been investigated and different recommendations on drug selection have been made. One of the frequently used regimens is oral administration of a flouroquinolone 30-60 min before biopsy which is continued for 2-3 days after biopsy. Although minimal infectious complications occur with the existing prophylaxis protocols the 0.1-0.5% rate of bacteremia or sepsis is still an important problem (3). In this study the efficacy of the duration of prophylactic antibiotic administration in patients undergoing transrectal ultrasound (TRUS) guided biopsy was evaluated.

## MATERIALS AND METHODS

The study was initiated after the approval of local ethics committee. The data of patients undergoing a prostate biopsy between September 2007 and June 2009 with a suspicion of prostate cancer were retrospectively reviewed. A total of 367 patients with complete records were included. Patients with a positive urine culture before the biopsy, a history of urologic intervention in the last 3 months, immunodeficiency state and catheterized patients were excluded. Patients were divided into 2 groups according to the duration of antibiotic prophylaxis they received. Oral prophylactic antibiotics were administered to all patients before biopsy. Patients receiving oral ciprofloxacin (750 mg every 12 hours) for 3 or more days were assigned to Group-1 and for single day to Group-2.

In our clinic prostate biopsy is offered to patients who have positive findings in digital rectal examination and/or a total PSA level >2.5ng/mL. Routinely 10-12 cores biopsies are taken. Additional cores are also included in case of any suspicious area. All biopsies are performed by using 7.5 MHz transrectal probe and its fitting attachment of Sonoscape SSI-2000 BW system (Sonoscape. co. Ltd.) ultrasound device. The anticoagulation treatment is discontinued 1 week before the biopsy. Bowel preparation, dietary modifications and local area preparation are not performed routinely. The first dosage of the prophylactic antibiotic was administered orally 30-60 min before the procedure. The second dose was suggested to be taken 12 hours after biopsy.

Standard patient forms including biopsy related complications were used in biopsy unit of our clinic. Demographic characteristics of patients, symptoms, PSA values, IPSS scores, prostate sizes, pathologic results and complications occuring after biopsy were recorded in those forms. Haematuria was defined as persisting bleeding for more than 24 hours after biopsy. Fever was defined as the presence of any temperature higher than 38ºC in the first week after biopsy without any local infection findings and with negative urine culture. Acute prostatitis was defined as fever higher than 39ºC dysuria, frequency, perineal pain or discomfort with positive urine culture.

Acquired data were transfered to a computer environment and statistical analysis was performed by software. The results were expressed as mean ± standard deviation. Categorical and continous variables were analysed with Chi-square and Student T tests respectively. A p value of <0.05 was considered statistically significant.

## RESULTS

Of 367 patients 243 were classified as Group-1 and the rest 124 were grouped as Group-2. The mean age of all patients was 63.92 years and the mean PSA was 13.61ng/dL. The pre-biopsy mean IPSS score was 12.47 and mean prostate volume 52.53 mL. For 78.2% of patients the current biopsy was their first biopsy. Cancer detection rate was 24.2%. These characteristics for each group are shown in Table-1. There was no significant difference between groups in terms of age, first biopsy rate, PSA values, IPSS, prostate volume and cancer detection rates. In terms of complications, hematuria was recorded in 25 (10.3%) patients in Group-1 and 27 (21.8%) patients in Group-2 (p=0.003). Fever was observed in 3 (1.2%) patients in Group-1 and 5 (4.0%) patients in Group-2. While comparing local infection (epididimitis, orchitis, etc.) rates in Groups 1 and 2, local infections occurred in 2 (0.8%) and 1 (0.8%) patients respectively in Groups 1 and 2. Acute prostatitis was not observed in any patients in Group-1 and was observed in only 1 (0.8%) patient in Group-2. None of the patients developed septicemia or other serious infection. Only in one patient hospitalization due to acute prostatitis was required. When the two groups were evaluated according to their infectious complications there was no statistically significant difference in terms of fever, local infections (epididimitis, orchitis, etc.) and acute prostatitis. The complication rates in the two groups are shown in Table-2.

**Table 1:** Patient characteristics in the two groups

	Total	Group-1	Group-2	P value
Number of patients	367	243	124	-
Age	63.92 ± 7.18	63.67 ± 7.06	64.41 ± 7.42	0.353
(range)	(48-83)	(46-79)	(48-85)	
First biopsy rate (%)	287 (78.2)	190 (78.2)	97 (78.2)	0.553
PSA (ng/dL)	13.61 ± 20.04	13.15 ± 19.84	14.51 ± 20.47	0.537
(range)	(2.77-130)	(2.77-121)	(3.04-130)	
IPSS	12.47 ± 7.82	12.74 ± 7.76	11.94 ± 7.95	0.377
Prostate volume (mL)	52.53 ± 32.30	51.68 ± 32.31	54.04 ± 32.35	0.515
(range)	(11.25-191.89)	(11.25-173.56)	(18.76-191.89)	
Cancer detection rate (%)	88 (24.2)	56 (23.2)	32 (26.2)	0.838

Mean ± SD

**Table 2:** Complication rates among groups.

	Total	Group-1	Group-2	P value
Number of patients	367	243	124	–
Hematuria (%)	52 (14.2)	25 (10.3)	27 (21.8)	0.003
Fever (%)	8 (2.2)	3 (1.2)	5 (4.0)	0.090
Local infections (%) (epididimitis, orchitis, etc)	3 (0.8)	2 (0.8)	1 (0.8)	0.735
Prostatitis (%)	1 (0.3)	–	1 (0.8)	0.338

## DISCUSSION

Transrectal biopsy is frequently performed nowadays and it is regarded as a relatively safe procedure in general. But, owing to its traumatic nature this procedure may cause hemorrhagic (hematuria or rectal bleeding) and infectious complications. Infectious complications may be in the form of asymptomatic bacteriuria as well as it can present as urinary infections, fever or sepsis. It can even cause death following septicemia (4). American Urology Association recommends single dose antibiotic prophylaxis before prostate biopsy (5). Currently prophylaxis with quinolones is still recommended despite increased rate of infectious complications (5).

In a study conducted in 144 hospitals in England and Ireland (6) the use of 13 antibiotics in 48 different regimens was reported. They suggested ciprofloxacin or norfloxacin regimens as inexpensive and efficient. An evaluation of 88 different centres in USA has revealed that 83% of patients received antibiotic prophylaxis before biopsy and in 81% of these cases quinolones were used (7). One of the most frequently used protocols is to administer a quinolone orally 30-60 min before the biopsy and continue for 2-3 days after the biopsy. In spite of this it was reported that no hospital admissions due to febrile urinary tract infection were required with the use of a single dose of ciprofloxacin (8).

In a randomized controlled trial, Aron et al. (9) divided 231 patients into 3 groups: Group-1 (n=75) received placebo twice daily for 3 days, Group-2 (n=79) received single doses of ciprofloxacin 500 mg and tinidazole 600mg and Group-3 (n=77) received the same treatment as Group-2 for 3 days. The complications were classified as infectious or noninfectious. Noninfectious complications were defined as lower urinary tract symptoms, rectal bleeding for more than 12 hours, hematuria for more than 12 hours and perineal pain. Infectious complications included fever (body temperature>38°C), urinary tract infection, prostatitis, fever+urinary tract infection and prostatitis+fever+urinary tract infection. When complications rates were compared between the two groups, infectious complications were significantly higher in the placebo group compared to single dose and 3-day treatment groups. The groups were similar in terms of fever and bacteremia while there was a significant difference in terms of urinary tract infections. At the end of the study hematuria was present in 8 of 75 (10.7%), 13 of 79 (16.5%) and, 12 of 77 (15.6%) patients in Groups 1, 2 and, 3 respectively. Our results showed a significant difference between the groups in terms of hematuria while Aron et al. did not find such a difference. It may be a result of the fact that mean number of cores were not the same in both groups. Patients with elevated PSA (>20ng/dL) were biopsied with fewer cores. By using single dose prophilaxy safely during the period more cores were taken routinely (upgraded 12 from 10). Aron et al. detected fever in 5, 2 and 2 of patients in Groups 1, 2 and 3 respectively. In our study we detected fever in 3 and 5 of the patients in Groups 1 and 2 respectively. In both of these studies there was not a statistically significant difference in terms of fever detection. When these two studies are evaluated in terms of prostatitis, while Aron et al. reported prostatitis in 6, 8 and 3 of patients in each of the three groups we have found prostatitis in only one patient in Group-2. A statistically significant difference in prostatitis rates between groups were reported in both studies. Aron et al. concluded that antibiotic prophylaxis was mandatory in prostate biopsy and prophylaxis for 3 days was superior to single dose prophylaxis.

Also Cam et al. (10) divided 400 patients into three groups in a randomized study to show that a single dose of antibiotic is adequately effective in prophylaxis. Patients were administered 1 gr of intramuscular ceftriaxone single dose, oral ciprofloxacin 500 mg twice daily for 3 days and oral ciprofloxacin 500 mg single dose in Groups 1, 2 and 3 respectively. Their results, similar to our study showed no significant difference in terms of infectious complications between the 3 groups.

Shigemura et al. (11) evaluated a total of 236 patients in two groups in a randomized study to prove the efficacy of single day prophylaxis for prostate biopsy. One hundred twenty four patients in Group-1 received 600 mg levofloxacin once daily and 112 patients in Group-2 received levofloxacin 300 mg daily for 3 days. They observed febrile infectious complications in 2 patients in each group. Their results showed no significant difference in terms of febrile complications between the groups, similar to our study.

Briffaux et al. (12) treated 139 patients with 2 tablets of ciprofloxacin 500mg 2 hours before the biopsy and 149 patients with the same dose of ciprofloxacin for 3 days, to prove the efficacy of single dose in prophylaxis. They observed asymptomatic bacteriuria in 6 patients and prostatitis in 1 patient in both groups. In line with our results there was no signifant difference in the post-biopsy symptom scores at both groups. The groups were similar in terms of complication rates.

Sabbagh et al. (13) administered a fluoroquinolone for durations of 1 and 3 days as a prophylaxis before prostate biopsy in a randomized study. They did not find significant difference in complication rates.

In a study of Aslan et al. (14) the febrile and non febrile urinary infection rates were reported to be similar with treatment regimens of single dose levifloxacin and single dose fosfomycin+500 mg of ciprofloxacin twice daily for 5 days. But they observed higher rates of fluorochinolone resistant and extended spectrum beta lactamase (ESBL) resistant E.Coli in urine cultures of those patients receiving ciprofloxacin.

Taylor et al. (15) obtained urine and rectal swab cultures of patients before biopsy in his prospective study. In 19% of 865 patients ciprofloxacin resistant gram negative coliform bacteria was detected. In 90.6% of these the bacteria were E.Coli. The high risk factors for developing resistance were a history of using ciprofloxacin in the last 3 months and surgery relating to cardiac valve diseae. Infectious complications were detected in 3.6% (n=31) of patients. In 48% of these ciprofloxacin resistant organisms were detected in rectal swabs. Among patients who had positive rectal swab cultures for ciprofloxacin resistant bacteria 9% developed infectious complications. Despite a positive rectal swab culture for ciprofloxacin resistant E.Coli, infectious complications did not occur in those patients receiving ciprofloxacin prophylaxis. Since rates of infectious complications were similar in single dose and longer prophylaxis with ciprofloxacin, the most appropriate prophylactic regimen was suggested as single dose applications despite increasing rates of resistance.

The major limitations of the current study are its retrospective design and absence of data regarding antibiotic usage in the last 3 months. More Urologists are performing prostate biopsy in their daily practice. Antibiotic resistance and infectious complications are increasing throughout the World and especially in our country. We tried to reflect the results of our daily practice to navigate clinicians more antibiotic is not resulting low infectious complications.

## CONCLUSIONS

In a selected patient population single dose prophylaxis with ciprofloxacin can be safely administered compared to other regimens of 3 days or more duration. Increasing the duration of antibiotic prophylaxis does not decrease infectious complications. Nevertheless it should be kept in mind that there is always some risk of infectious complications. Single dose prophylaxis is easy to administer and reduces costs.
